# Multi-scale structural similarity embedding search across entire proteomes

**DOI:** 10.1101/2025.02.28.640875

**Published:** 2025-03-06

**Authors:** Joan Segura, Ruben Sanchez-Garcia, Sebastian Bittrich, Yana Rose, Stephen K. Burley, Jose M. Duarte

**Affiliations:** 1 Research Collaboratory for Structural Bioinformatics Protein Data Bank, San Diego Supercomputer Center, University of California San Diego, La Jolla, CA 92093, USA; 2 School of Science and Technology, IE University, Paseo de la Castellana 259, 28046 Madrid, Spain.; 3 Research Collaboratory for Structural Bioinformatics Protein Data Bank and the Institute for Quantitative Biomedicine, Rutgers, The State University of New Jersey, Piscataway, NJ 08854, USA; 4 Rutgers Cancer Institute, Rutgers, The State University of New Jersey, New Brunswick, NJ 08901, USA; 5 Department of Chemistry and Chemical Biology, Rutgers, The State University of New Jersey, Piscataway, NJ 08854, USA; 6 Rutgers Artificial Intelligence and Data Science (RAD) Collaboratory, Rutgers, The State University of New Jersey, Piscataway, NJ 08854, USA

## Abstract

The rapid expansion of three-dimensional (3D) biomolecular structure information, driven by breakthroughs in artificial intelligence/deep learning (AI/DL)-based structure predictions, has created an urgent need for scalable and efficient structure similarity search methods. Traditional alignment-based approaches, such as structural superposition tools, are computationally expensive and challenging to scale with the vast number of available macromolecular structures. Herein, we present a scalable structure similarity search strategy designed to navigate extensive repositories of experimentally determined structures and computed structure models predicted using AI/DL methods. Our approach leverages protein language models and a deep neural network architecture to transform 3D structures into fixed-length vectors, enabling efficient large-scale comparisons. Although trained to predict TM-scores between single-domain structures, our model generalizes beyond the domain level, accurately identifying 3D similarity for full-length polypeptide chains and multimeric assemblies. By integrating vector databases, our method facilitates efficient large-scale structure retrieval, addressing the growing challenges posed by the expanding volume of 3D biostructure information.

## Introduction

Recent advances in deep neural architectures have revolutionized large-scale prediction of 3D structures of biological macromolecules. Deep learning models^1,2^, most notably AlphaFold2^3,4^, have demonstrated remarkable success in predicting protein structures with accuracies comparable to that of lower-resolution experimentally determined structures^5^, significantly accelerating structural biology research^6,7^. More recently, models like the Evolutionary Scale Modeling 3 (ESM3)^8^ have further expanded the horizon of applications by leveraging large-scale protein language models (PLM) to predict both structure and function. These manifold breakthroughs have led to the creation of extensive structural databases, such as AlphaFold DB^9^, which now hosts more than 214 million structures predicted by Google Deep Mind, ESMAtlas^10^, a database of over 600 million protein structures derived from metagenomic sequences, and the ModelArchive^11^, a repository for computed structure models open to all contributors. By providing high-quality structural data at an unprecedented scale, these advances are transforming protein structure analysis and reshaping computational approaches to process structural information efficiently.

The Protein Data Bank (PDB)^12^ serves as the global repository for experimentally determined macromolecular structures, providing an essential resource for structural biology, structure-guided drug discovery, and structural bioinformatics. Jointly managed by the Research Collaboratory for Structural Bioinformatics Protein Data Bank (RCSB PDB, RCSB.org) in the United States, together with its Worldwide Protein Data Bank (wwPDB, wwpdb.org) partners, the PDB has long been the gold standard for high-resolution 3D biostructure data generated by macromolecular crystallography, 3D electron microscopy, and nuclear magnetic resonance spectroscopy. Importantly, the success of deep learning-based structure prediction methods—such as AlphaFold2, RoseTTAFold, and ESM3—would not have been possible without the vast repository of rigorously validated, expertly biocurated experimental structures stored in the PDB. PDB data were essential for training these and other AI/DL models. Recognizing the growing impact of deep learning prediction methods, RCSB.org has expanded its research-focused web portal to deliver computed structure models (CSMs)^13^, including those generated by AlphaFold2 and those deposited into the ModelArchive. Currently, RCSB.org hosts >1 million CSMs, providing open access to the entire proteomes of human, model organisms, agriculturally important plants, select bacterial pathogens, and species relevant to understanding and mitigating climate change^14^. By incorporating these predicted structures alongside experimentally determined structures, RCSB.org provides many millions of researchers, educators, and students with a comprehensive one-stop shop for 3D biostructural analysis, functional annotation, and drug discovery. RCSB.org bridges the gap between experimentally validated data and large-scale predictive modeling, facilitating new avenues for understanding protein function, evolution, and biologically important molecular interactions at unprecedented scale.

As the volume of available 3D biostructure data continues to grow, efficient searching and comparisons among structures at scale have become critical challenges for computational structural biologists. With resources such as AlphaFold DB and the ModelArchive hosting hundreds of millions of predicted protein structures, and RCSB.org expanding its catalog of integrated CSMs, structural similarity search methods must support real-time, accurate queries across a vast and continuously growing 3D biostructure space. Moreover, structure comparisons are inherently complex due to the multi-scale nature of macromolecular architecture (spanning primary, secondary, tertiary, and quaternary levels). Macromolecular structures can be analyzed at different hierarchical levels of granularity, including individual domains, full-length protein chains, and larger multimeric assemblies, each of which may exhibit unique structural and functional properties. Effective search algorithms must account for these varying levels of granularity while maintaining computational efficiency, ensuring that relevant structural relationships can be identified across diverse datasets. Developing scalable and precise structural similarity search methods is, therefore, essential for unlocking new biological insights and enabling the functional annotation of uncharacterized proteins and assemblies within the rapidly expanding corpus of structural information.

Traditional structure superposition tools^15,16^ are computationally complex, rendering them unsuitable for large-scale, real-time similarity searches. To improve search performance, bioinformaticians have relied on large-scale computational resources to handle computationally intensive steps^17^ or previous clustering based on structure to reduce the search space^18^. Both such approaches have limited scalability.

Recently developed methods offer various solutions for searching large volumes of structural data while aiming to retain high levels of sensitivity. One such approach, BioZernike descriptors^19^, encodes macromolecular structures as vectors of features using Zernike polynomials and global geometric measures. A logistic regression model, trained using the CATH classification as ground truth, is then applied to compare pairs of descriptors. Although this method efficiently handles the current volume of experimental structural data, its sensitivity is lower compared to other approaches.

A complementary approach, Foldseek^20^, transforms 3D protein structures into sequences using a structural alphabet, effectively reducing the structural search problem to a sequence similarity search task. When integrated with the sequence search software MMseqs^21^, Foldseek enables real-time searches in large datasets, provided that the data is clustered and sufficient computational resources are available. Currently, Foldseek is employed as the structural search method in the AlphaFold DB server, utilizing a pre-clustered subset of the 214 million structures at 50% sequence identity to improve performance.

Embedding-based approaches transform complex data—such as text, audio, and images—into high-dimensional vector representations that capture meaningful relationships in a continuous space. Such embeddings enable efficient similarity searches by positioning similar entities closer together in the vector space, allowing for fast and scalable comparisons. To manage the growing volume of high-dimensional data, vector databases store and index these embeddings, enabling large-scale retrieval across millions of entries, by employing Approximate Nearest Neighbors (ANN) algorithms. This approach has revolutionized multiple fields, including natural language processing^22^ and computer vision^23^. In computational biology, the TM-Vec approach applies this concept to protein sequences, encoding them as vectors to facilitate remote homology detection^24^.

Herein, we present a scalable structural similarity search strategy designed to traverse enormous volumes of 3D biostructure data, including both experimentally determined atomic coordinates and CSMs. Our approach follows an embedding-based methodology, transforming macromolecular structures into fixed-length vectors and leveraging vector databases for optimal search performance. The method integrates the ESM3 generative model to convert 3D structures into sequences of residue embeddings, which are then processed by a transformer-based network that aggregates the information into a fixed-length vector. The embedding model was trained to predict TM-scores between pairs of 3D structures. Despite being trained exclusively on single-domain structures, our results demonstrate that the model generalizes beyond the domain level to full-length polypeptide chains (frequently encompassing multiple structurally dissimilar domains). Moreover, we extended the embedding representation to macromolecular assemblies, achieving competitive performance in structure similarity searches for multi-protein complexes. Finally, we set up a proof-of-concept system that stores embeddings for 214 million CSMs from AlphaFold DB in a vector database, showing the immediate feasibility of handling the current structural knowledge with modest computational resources. The system has been made publicly available at http://embedding-search.rcsb.org, allowing queries for PDB and AlphaFold DB entries or custom user-uploaded files.

## 2 Materials and Methods

### 2.1 Datasets

This section introduces the various datasets used for training and testing our embedding model. Domain pairs training set: The training set consists of 15,176 SCOPe40 (v2.08)^25^ domains from the ASTRAL subset, a non-redundant collection with <40% sequence identity, corresponding to protein domains identified in PDB structures. These domains represent 4,703 families and have an average length of 182 amino acids. The dataset was compiled by Chengxin Zhang, and all associated information, including domain coordinate files, is publicly available in the Zenodo repository for research purposes^26^. Both TM-scores, query, and target, are available for all domain pairs, resulting in over 115 million comparisons. For training, we used the highest TM-score in each pair. This dataset of domain pairs and TM-scores will be referred to as DT115M hereafter.

#### Domain pairs benchmarking:

To evaluate the performance of the embedding model for structural similarity search and compare it to results obtained with Foldseek and other structural search methods, we used the benchmark described in reference^20^. This dataset consists of 11,211 single domains from SCOPe40 (v2.01), with an average length of 174 residues, representing 4,161 distinct families (hereafter DS62M). All domain pairs (>62 million) in the dataset were used to assess the capability of each method to identify matches at the family, superfamily, and fold levels.

#### Single domain chains:

A third protein domain dataset was used to study the latent information encoded within the embeddings. This dataset consists of a non-redundant subset of CATH domains^27^ (CATHS40), where sequences share less than 40% identity, excluding domains longer than 300 residues (hereafter DS29K). The dataset includes 29,735 domains of an average length of 136 amino acids and representing 5,896 homology superfamilies.

#### Full-length chain benchmarking:

A non-redundant set of full-polypeptide chain protein structures was harvested from the PDB. We used RCSB.org sequence clusters to guarantee an identity level of less than 30% between proteins. Subsequently, only proteins with sequence lengths longer than 200 amino acids and fewer than 20 structurally unmodelled residues were considered for the final collection resulting in 7,899 protein chains. Finally, we computed the TM-score value for all possible pairs using the US-align software enabling the ‘-fast’ configuration option (hereafter PS31M). The resulting dataset contained more than 31 million protein pair comparisons. This dataset was used to evaluate the performance of our approach for structural similarity searches in full-polypeptide chain protein cases longer than the average single domain lengths used in the other benchmarks.

#### Assembly Dataset:

To evaluate the performance of our embedding model for structural similarity searches in multimeric protein assemblies, we used a dataset similar to the one described in the Foldseek-Multimer publication^28^. This collection of structures was compiled from the 3DComplexV7 database^29^, considering homomeric assemblies with 2 to 24 subunits. For each assembly size, all pairs with a TM-score greater than 0.8 (as computed by Kpax^30^) and sequence identity below 80% between their monomers were selected. When more than 100 pairs met these criteria, only the first 100 hits were retained. This process resulted in a total of 931 assembly pairs involving 677 different assemblies.

To complete our assembly evaluation dataset, we conducted an all-vs-all comparison among the 677 assemblies using the US-align software, computing the TM-score for all 228,826 possible pairs, including those with different numbers of subunits (hereafter AS228K).

### 2.2 Embedding Model

**Figure 1.**
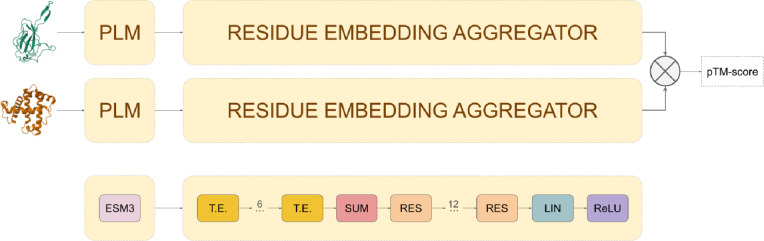
Embedding model network architecture. The model operates as a twin neural network, utilizing shared weights to calculate embeddings for pairs of 3D structures and predict TM-scores from their cosine distance. The model consists of two main components: (1) a protein language model (PLM), ESM3, which encodes protein 3D structures as residue-level embeddings, and (2) an aggregator module that processes these embeddings into a fixed-length vector. The aggregator comprises six transformer encoder layers (T.E.), a summation pooling operation (SUM), and twelve fully connected residual blocks (RES).

The embedding model transforms protein structures into numerical vectors of fixed length. The model consists of two main components: (1) a PLM that computes an embedding for each residue in a given 3D structure, and (2) a transformer-based neural network that aggregates these residue-level embeddings into a single vector, encoding information about the entire 3D structure. Figure 1 displays a visual representation of the embedding model and its different components.

Residue-level embeddings are computed using ESM3, a generative PLM^8^. Given the 3D structure of a single protein chain, this model outputs a 1536-dimensional vector for each residue, capturing both sequence and structural information.

The aggregation network processes the residue-level embeddings from ESM3 and outputs a fixed-length vector. This component consists of six stacked transformer encoders^31^, each with feedforward layers containing 3,072 neurons and ReLU activations. Following the transformer encoders, a summation pooling operation and 12 fully connected residual layers^32^ aggregate the resulting embeddings into a single 1,536-dimensional vector, encoding information for the entire 3D structure.

### 2.3 Model Training

Our embedding model was trained to predict the maximum TM-score between pairs of 3D structures. TM-score values were rounded to the first decimal place allowing the network to approximate 11 possible TM-score values:

$$n\times 10^{-1}\;\text{for}\;n=0,\ldots ,10.$$

During training, the model operated as a twin neural network, utilizing shared weights to produce embeddings for pairs of 3D structures (see Figure 1). The ESM3 model weights are frozen and the aggregator network parameters were optimized to minimize the mean square error between the cosine similarity of the computed embeddings and the TM-score of their superposed 3D structures.

**Figure 2.**
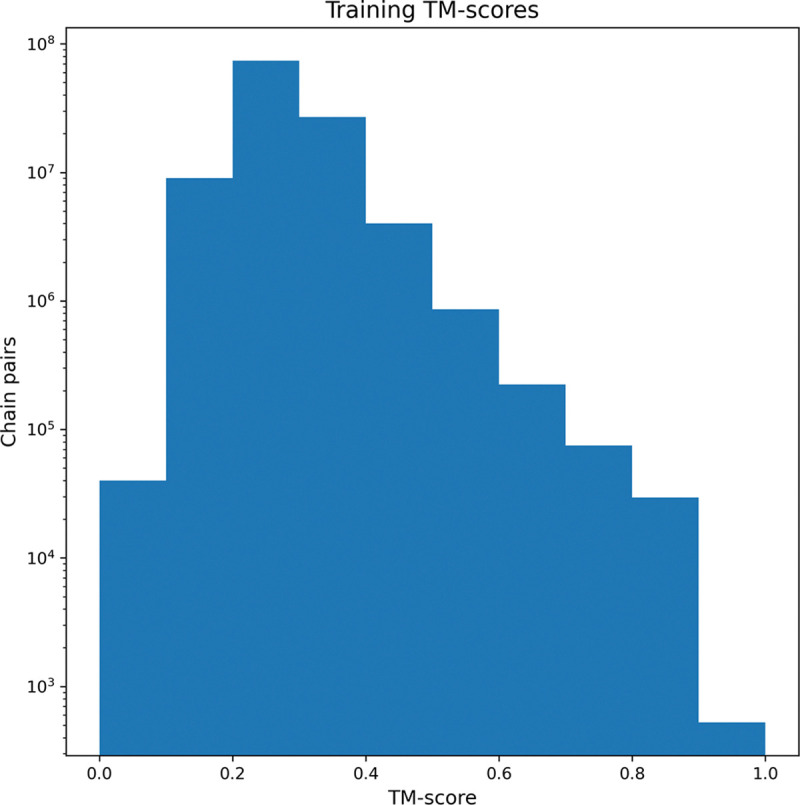
TM-score distribution for the SCOPe40 v2.08 training pairs. Histogram of TM-scores for domain pairs (>115M) used in training, illustrating an imbalanced distribution. The vast majority (>95%) of TM-scores are below 0.4, while fewer than 0.05% exceed 0.8.

The final model was trained using domain pair batches from the DT115M dataset. Batches were randomly generated ensuring that the TM-score values of the domain pairs were uniformly distributed among the 11 possible values. This strategy addresses the unbalanced nature of TM-score values within the DT115M dataset, where only 0.05% of TM-score values exceed 0.8 and more than 95% fall under 0.4 (see Figure 2).

To evaluate model performance and store checkpoints during the training process, a validation dataset was created by removing 2% of the DT115M training dataset. After each epoch, the area under the precision-recall curve (AUPRC) was calculated on the validation set as a measure of performance. The model was trained for 100 epochs, each consisting of 320,000 randomly selected domain pairs. The best performance was achieved at epoch 31, with no significant improvement thereafter. We used the Adam optimizer^33^ with a learning rate schedule incorporating cosine decay and a two-epoch warmup. Training was conducted using 64 A100 GPUs.

### 2.4 Assembly embeddings

We extended our model to predict embeddings for multimeric assemblies using the following strategy. For a given assembly, the generative model ESM3 was used to compute residue-level embeddings for each chain individually. Since ESM3 operates at the chain level, the resulting per-residue embeddings are independent of the chain order in the assembly. Then, chain residue-level embeddings were concatenated to form a single sequence of embeddings. Subsequently, our residue-level aggregator model was applied to compute a fixed-length vector representation for the whole 3D structure of the assembly. Since the aggregator module does not include any positional encoding, it remains equivariant to chain permutations, and when combined with the summation pooling operation, this ensures that the final embedding representation is invariant to the selected chain order in the assembly.

Although our model was trained exclusively on single domains, Section 3.4 demonstrates how the latent features learned by our embedding model generalize beyond single-domain structures, enabling it to perform structural similarity searches at the assembly scale.

### 2.5 Structural Embedding Database

A dedicated vector database (Milvus) was deployed to store embeddings for all structures integrated into RCSB.org, including both experimental entries and CSMs. More than 2 million embeddings were computed, covering all individual chains and biological assemblies, and subsequently indexed using the Hierarchical Navigable Small World (HNSW) algorithm to ANN searches^34^. The HNSW algorithm constructs multi-layered graphs that efficiently balance speed and accuracy, significantly reducing search complexity for large-scale vector datasets. To demonstrate the scalability of the embedding approach, we generated embeddings for all predicted structures (>214 million) available in AlphaFold DB^9^ and stored them in a separate vector database. Due to the large data volume, the DiskANN indexing approach^35^ was used to create a disk-based index while maintaining efficient memory use and search speed.

## 3 Results

### 3.1 Sensitivity benchmark

Our primary objective in developing this new embedding model is to identify 3D structures similar to a user-provided query. To evaluate its performance and compare it with Foldseek and other methods described in reference^20^, we utilized the same evaluation benchmark based on the DS62M dataset. An all-versus-all domain comparison was performed, and for each domain, we measured the sensitivity as the fraction of true positives (TPs) detected up to the first false positive (FP). This benchmark effectively simulates the scenario wherein a user expects to retrieve all the structures similar to a given query before encountering the first negative or weakly related case.

The performance was assessed at the domain family, superfamily, and fold recognition levels. For all three levels, TPs were defined as follows: pairs sharing the same family, pairs sharing the same superfamily but not the same family, and pairs sharing the same fold but not the same superfamily, respectively. Domains from different folds were classified as FPs. For each domain query, results were ranked from highest to lowest score ignoring self-comparison, and sensitivity to the first FP was calculated as the fraction of TPs.

**Figure 3.**
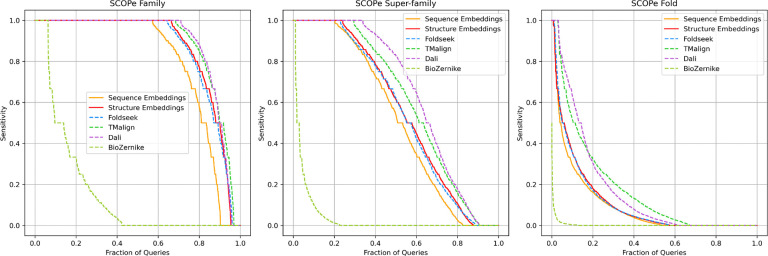
Sensitivity benchmark for family, superfamily, and fold detection on SCOPe40 v2.01 domain structures. Each domain was used as a query and compared against all other domains in the dataset. Results were ranked in descending order of predicted similarity score. True positives (TPs) were defined as matches within the same family, within the same superfamily but different families, and within the same fold but different superfamilies. False positives (FPs) were matches between different folds. Sensitivity was assessed by counting the number of TPs retrieved before the first FP. “Sequence Embeddings” were computed using only sequence-derived features, whereas “Structure Embeddings” integrated both sequence information and structural coordinates.

Figure 3 illustrates sorted sensitivity values, ranging from the best- to lowest-performing queries. Although our embedding model does not reach the sensitivity of more computationally intensive structural alignment methods (e.g., DALI and TM-align), it performs as well as Foldseek and significantly outperforms our earlier BioZernike descriptors approach. Additionally, Figure 3 demonstrates that using structural information for residue-level embeddings provides superior performance versus relying solely on sequence information.

Since SCOPe domain families in the DS62M benchmark dataset overlap with the DT115M training set, the benchmark described above was evaluated using a 10-fold cross-validation strategy to prevent redundancy between training and testing. The DS62M dataset was divided into 10 distinct testing subsets, each containing a unique collection of SCOPe families. For each testing subset, an embedding model was trained with all SCOPe families from the corresponding testing subset excluded from the training data. This precaution ensures that the training set contains no information about the domain queries used during evaluation. Importantly, the training procedure is based on the prediction of TM-scores between protein domain pairs, and no information from the SCOPe classification is explicitly included.

### 3.2 Structural information in the latent space

We used the DS29K dataset to analyze the latent representations produced by our embedding model in relation to the CATH classification of domains. For each level in the CATH (Class, Architecture, Topology, and Homology) hierarchy, we selected all domains belonging to the five most populated categories. Embeddings for these domains were computed using our model, and t-SNE (t-distributed Stochastic Neighbor Embedding) was applied to reduce their dimensionality and project them into a 2D space. Different color schemes based on the CATH categories were used to visualize whether the embeddings of domains from the same category clustered together.

**Figure 4.**
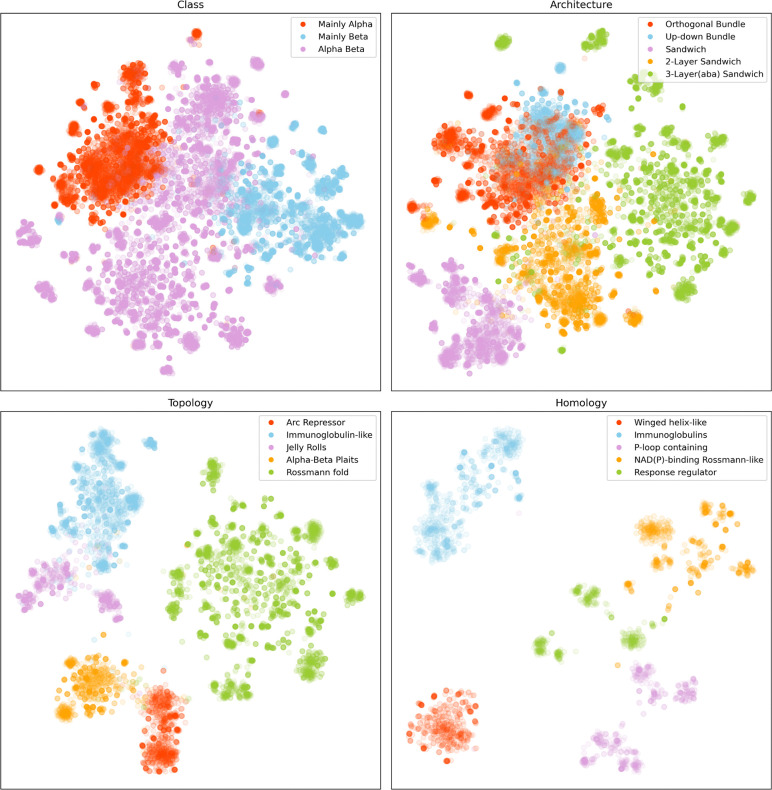
Latent information analysis of CATH40 domain embeddings. t-SNE dimensionality reduction of domain embeddings from the five most represented categories at each CATH hierarchical level (Class, Topology, Architecture, Homology). The embeddings capture structural features that enable clear separation of domains according to their classification across different levels.

Figure 4 shows a 2D projection of selected CATH domains at the different hierarch levels. In each case, domains belonging to the same category are clustered together. For example, in Figure 4 (class), domains are colored based on their class: mainly alpha (pink), mainly beta (blue), and alpha/beta (purple). The alpha and beta domains form two distinct clusters, with the alpha/beta domains positioned in between as expected. These results demonstrate that the information learned by our model implicitly encodes representations that strongly align with the hierarchical classification of protein domains defined in CATH.

### 3.3 Full-length chain Benchmark

In our previous benchmarks, we used domain-level datasets to assess the performance of our embedding model in identifying structural similarity. However, a more common scenario involves finding structures similar to a given protein chain. To demonstrate that our approach extends beyond the domain-level granularity used during training, we evaluated its performance on the PS31M dataset, a non-redundant collection of full-polypeptide chain proteins extracted from the PDB.

**Figure 5.**
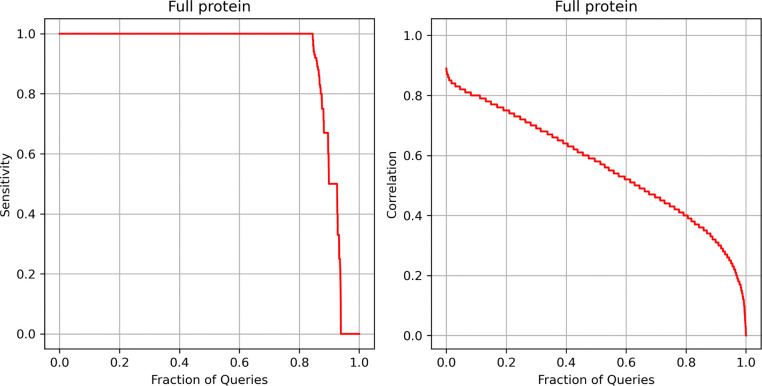
Sensitivity and Pearson correlation results for a non-redundant set of PDB proteins. Each protein was used as a query and compared against the rest of the dataset. True positives (TPs) were protein pairs with a TM-score greater than 0.8, while false positives (FPs) had a TM-score lower than 0.5. Sensitivity was measured up to the first FP. The correlation between predicted and computed TM-scores was calculated for each protein and ranked from best to worst.

We performed a sensitivity test similar to that described in Section 3.1. In this benchmark, TPs were defined as protein pairs with a TM-score greater than 0.8, while FPs were those with a TM-score below 0.5 (proteins of distinct fold^36^). Each protein was compared against the entire dataset, and the results were ranked from best to worst predicted score. As in Section 3.1, sensitivity to the first FP was then calculated. Figure 5 illustrates sensitivity as a function of the fraction of queries (the fraction of proteins in the dataset). For more than 85% of the proteins in the PS31M collection, our approach achieves 100% sensitivity, meaning it successfully retrieves all protein pairs with a TM-score greater than 0.8 before encountering the first FP (TM-score < 0.5).

**Figure 6.**
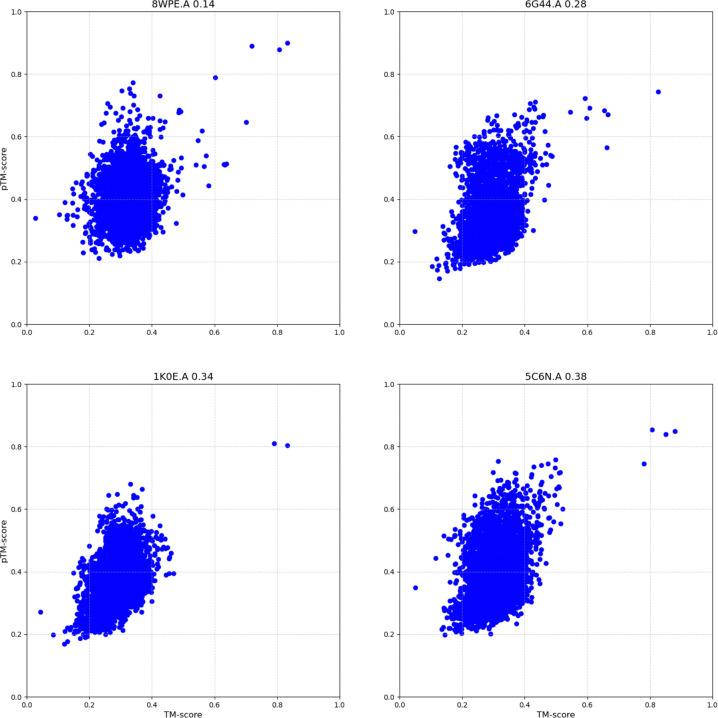
Predicted vs. computed TM-scores for four different PDB chains. Plot titles display the PDB chain and Person correlation value. Each chain was compared against the full dataset of non-redundant PDB chains and Pearson correlation was calculated between predicted and computed TM-score. Predicted scores exhibit a low correlation value but provide a strong sensitivity performance (100%).

**Figure 7.**
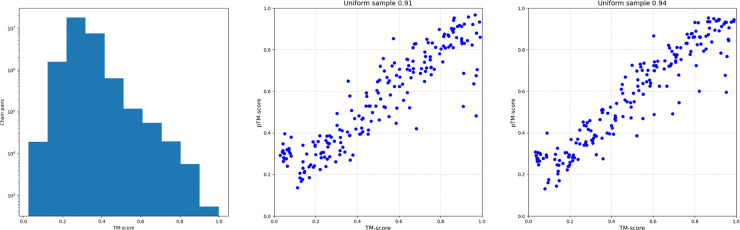
TM-score distribution for the PDB benchmarking pairs. The TM-score distribution is imbalanced within the unit interval, with 95% of values below 0.4. A strong correlation (~0.93) between predicted and computed TM-scores emerges when analyzing uniformly distributed random samples.

In addition to sensitivity, we calculated the Pearson correlation between the predicted and computed TM-scores for each protein when compared to the rest of the dataset. Figure 5 illustrates the relationship between correlation and the fraction of queries. For more than 80% of the proteins, correlation values exceed 0.4. However, we observed that correlation was not always a reliable measure of structural similarity search performance. For example, proteins represented by PDB chains 8WPE.A and 6G44.A exhibit a low correlation coefficient (<0.3; see Figure 6), yet still achieves 100% sensitivity to the first FP. The imbalanced distribution of TM-scores can lead to low correlation values when compared to predictions from our method, despite the predicted scores still providing strong recall. Figure 7 shows the distribution of TM-scores for the PS31M protein pairs, where 95% of the values range from 0 to 0.4. The correlation value calculated between computed and predicted TM-scores over the whole dataset is 0.6. However, when correlation is computed on a random sample uniformly distributed between 0 and 1, obtained values range from 0.91 to 0.94. Figure 7 presents several examples illustrating the high correlation between predicted and computed TM-scores for different uniformly distributed random samples.

The results obtained in this benchmark document that the embedding model learns beyond the original domain-based training set and generalizes well to full-polypeptide chain protein granularity for structural similarity searches.

### 3.4 Assembly Benchmark

Structural similarity search at the assembly level is a valuable operation that few open-access resources provide^28^. The primary goal of this benchmark is to evaluate the performance of our embedding model in identifying structures similar to a given multimeric assembly. Moreover, this test will demonstrate that the latent features learned by the embedding model extend to the quaternary structure of protein complexes, rather than being limited to single protein chains or domains.

In this benchmark, we used assembly structures included in the AS228K dataset and analyzed the relationship between sensitivity and the fraction of queries, following the same approach used in the protein benchmark (see Section 3.3). For each dataset assembly, we computed its embedding as described in Section 2.4. Then, each assembly was compared against the remainder of the dataset, and sensitivity to the first FP was calculated. Following the same criteria as used for the full-length chain benchmark, TP were defined as assembly pairs with a TM-score greater than 0.8, while FP were those with a TM-score lower than 0.5, as specified in the AS228K collection.

**Figure 8.**
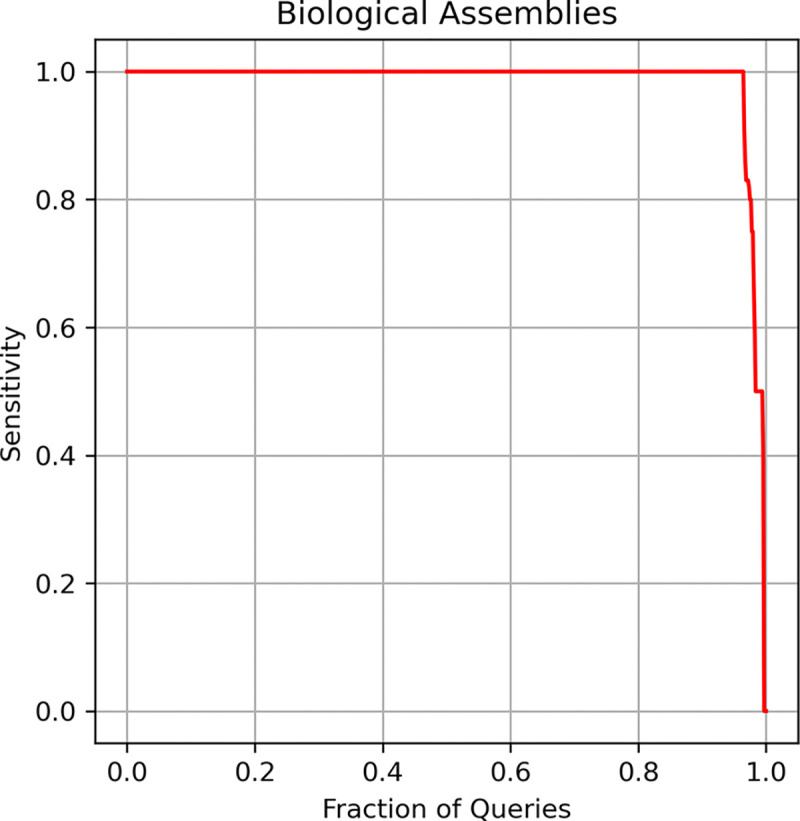
Sensitivity benchmark for a non-redundant set of PDB assemblies. Each assembly was used as a query and compared against the rest of the dataset. True positives (TPs) were assembly pairs with a TM-score greater than 0.8, while false positives (FPs) had a TM-score lower than 0.5. Sensitivity was measured up to the first FP.

Figure 8 shows that more than 95% of the assemblies (queries) achieve a sensitivity value of 100%, with only three cases out of 677 assemblies where no positive hits were found. This result indicates that the latent features learned by our embedding model effectively encode information at the assembly level, enabling strong performance in structural similarity searches of multi-protein complexes.

### 3.6 Scaling and Runtime

To assess the scalability and efficiency of our structural similarity search based on embeddings and vector databases, we evaluated its runtime performance across two datasets that represent extensive catalogs of protein structures: (1) all macromolecular structures from RCSB.org, which includes approximately 2 million chains from experimentally determined structures (from PDB) and CSMs (from AlphaFold DB and the ModelArchive), and (2) the 214 million predicted structures available from AlphaFold DB.

Both datasets were stored in independent Milvus database instances optimized for large-scale and fast vector retrieval. Chains from RCSB.org were indexed using the HNSW algorithm, and the database engine runs on a 32GB RAM, 8-CPU instance. The much larger AlphaFold DB dataset was indexed using DiskANN, a disk-based ANN method designed for large-scale retrieval with minimal memory overhead running on a 128GB RAM, 8-CPU instance. Search performance was evaluated by measuring query times as a function of the number of retrieved results. Due to the nature of ANN algorithms, retrieving a larger number of neighbors (results) requires additional graph index exploration, leading to increased query times. Table 1 presents the search times for different numbers of results returned per query and dataset.

As shown in Table 1, searches across all RCSB.org chains are essentially instantaneous, with average retrieval times of 0.02 seconds when returning 1000 results. Searches across AlphaFold DB exhibit longer runtimes, ranging from 0.43 seconds (10 results) to 8.95 seconds (1000 results). Despite this difference, the DiskANN-based approach remains highly efficient given the scale of the AlphaFold DB dataset, offering real-time structural retrieval capabilities across the equivalent of many thousand proteomes in their entirety.

Importantly, vector databases and ANN algorithms are an active field of research, so it is reasonable to expect more efficient systems to emerge^37^ to further improve the scalability and enable applicability to even larger datasets. It is also noteworthy that currently available vector database systems (e.g., Milvus, Elasticsearch, MongoDB) offer horizontal scalability capabilities, thus already allowing scale-ups to whole computer clusters.

## 4 Conclusion

In this work, we introduced a new structural similarity search strategy based on 3D structure embeddings and vector databases, capable of scaling to vast repositories of macromolecular structures, including both experimentally determined models and CSMs. Trained to predict TM-scores between single-domain structures, our approach demonstrated strong generalization beyond the domain level to full-length protein chains and even multi-protein assemblies. The embedding model achieved sensitivity levels comparable to those of state-of-the-art methods, while providing a highly scalable and computationally efficient alternative to traditional structural alignment techniques. Extensive benchmarking confirmed that our embedding model effectively captures structural similarities at multiple levels of granularity, aligning well with established protein domain classification databases. Moreover, its ability to retrieve structurally similar proteins and assemblies with high sensitivity highlights its potential for functional annotation, remote homology detection, and large-scale structural analysis. By integrating our method with a vector database infrastructure optimized for real-time retrieval, we enable efficient and scalable structural searches across expanding structural datasets, including AlphaFold DB with >214 million predicted structures, representing a significant step towards real-time, large-scale structural similarity searches for computational structural biology.

## Figures and Tables

**Table 1.** Query Performance Benchmark for Structural Similarity Search. Times are given in seconds.

Number of Results	RCSB.org chains (2M)	AlphaFold DB (214M)
10	0.003	0.43
100	0.004	1.14
1000	0.02	8.95

## Data Availability

https://zenodo.org/records/14933911
